# Iatrol

**Published:** 1893-10

**Authors:** E. D. Hayden


### TREATMENT OF WOUNDS BY IATROL.

In the case of a five-year-old mare, suffering from a severe cut on forefoot, caused by being caulked, I sprinkled the wound thoroughly once a day with Iatrol, and kept it bandaged. In four days it has entirely healed by first intention, and the hair grew again so that you could not discover that the foot had been injured. Since then I have used it in my many other cases of wounds, and invariably with good results. I consider it a most valuable antiseptic preparation for veterinarians, and had had such excellent results from its use that I should like to hear reports upon it from other practitioners.

E. D. Hayden, V. S.

